# Risk Factors and Psychosocial Correlates of Emotionally Negative Dreams in Patients Referred to a Cardiac Rehabilitation Centre

**DOI:** 10.21315/mjms2020.27.1.10

**Published:** 2020-02-27

**Authors:** Mozhgan Saeidi, Ali Soroush, Parvin Golafroozi, Ali Zakiei, Behrooz Faridmarandi, Saeid Komasi

**Affiliations:** 1 Cardiac Rehabilitation Center, Imam Ali Hospital, Kermanshah University of Medical Sciences, Kermanshah, Iran; 2 Lifestyle Modification Research Center, Imam Reza Hospital, Kermanshah University of Medical Sciences, Kermanshah, Iran; 3 Sleep Disorders Research Center, Kermanshah University of Medical Sciences, Kermanshah, Iran; 4 Department of Psychology, Islamic Azad University, Kermanshah, Iran; 5 Clinical Research Development Center, Imam Reza Hospital, Kermanshah University of Medical Sciences, Kermanshah, Iran

**Keywords:** anger, anxiety, cardiovascular disease, depression, dream, risk factors

## Abstract

**Introduction:**

Dream, as a kind of mental activity, includes various functions such as mood regulation, adjustment and integration of new information with the available memory system. The study was done for assessing the relationship between physiological and psychological components of cardiac diseases with emotionally negative dreams in cardiac rehabilitation.

**Methods:**

At the baseline of this cross-sectional study, 156 patients from Western Iran participated during April–November 2016. People 20 years–80 years able to recall the emotional content of dreams after cardiac surgery entered the study. The Beck depression inventory (BDI), Beck anxiety inventory (BAI), Buss and Perry’s aggression questionnaire (BPAQ) and Schredl’s dream emotions manual were used for collecting data. A binary logistic regression analysis used for the study of the relationship between risk factors and emotionally negative dreams.

**Results:**

The mean age of participants was 59 (SD = 9) years (men: 64.1%). The results showed that 25% of patients have negative emotional content. After adjustment for demographic variables, the results showed that increased anxiety [adjusted odds ratio (adj OR) = 1.08 [1.01–1.16], *P* = 0.020] and anger (adj OR = 1.03 [1.00–1.06], *P* = 0.024) and hypertension (adj OR = 2.71 [1.10–6.68], *P* = 0.030) can predict the dreams with negative content significantly.

**Conclusion:**

The increasing rates of anxiety and anger and history of hypertension are related to increasing dreams with the negative emotional load. The control of risk factors of dreams with negative emotional load can be the target of future interventions.

## Introduction

Dream, as a kind of mental activity that occurs during sleep (1), includes various functions such as mood regulation, adjustment, and integration of new information with the available memory system (2). Some theories suggest that physiological factors are the base of a dream and dreaming is fundamentally physiological driven (3). Mutually, other views believe that the role of psychological and behavioral factors is more important (2, 4, 5). These theories suggest that the content of a dream is concluded from cognitions and behaviour during the waking time (1, 5). So, dream probably has a role in the explanation of etiology and psychopathology in many psychiatric disorders (2, 4). For example, previous studies referred to the relationship between mood disorders such as depression (4), anxiety disorders (6) and alexithymia (7) with the contents of a dream.

The tensions during awaking time and occupational and social challenges through decreasing positive mood can lead to distressing sleep and annoying dreams (2, 8). For example, patients with cardiovascular problems usually experience high psychological distress because of death risk and invasive treatment procedures (9). Cardiac disease is concerned as a trigger to occur anxiety and depression or related disorders such as post-traumatic stress disorder (PTSD) (10–12). Anxiety disorders and depressed mood may be associated with negative dreams during sleep (4, 8).

Dreams have diagnostic and prognostic value and importance for therapeutic evaluation and assessment of psychiatric disorders, especially in mood disorders. The quantity and quality of dreams in patients with mood problems may predict remission from depression, response to antidepressants, and suicidal ideation and attempts (4). Thus, it seems that the assessment of dreams and their mechanisms and functions may help to percept cognitions, emotions, and complex behaviours in patients (2). Especially, in patients with cardiovascular problems, a study of dream mechanisms may help specialists predict the triggers and consequences of the illness (13).

Recent studies although have examined the relationship between dream and psychological and physical well-being included depression, insomnia, fatigue and other factors such as sex, age, the use of antidepressants or hypnotics and frequent heavy use of alcohol, cardiac symptoms among general population (14, 15), less focused on the population with cardiovascular problems (13). Thus, it seems that spontaneous study on physiological and psychological factors can facilitate the perception of complex mechanisms of the dream. We assume that both psychological and non-psychological factors influence the content of dreams, especially in patients with cardiovascular problems. The results of the current study will help to resolve the struggle and disputes of previous theories and studies (2–5). So, the present study aimed to assess the physiological and psychosocial correlates of dreams with negative emotional content in patients referred to cardiac rehabilitation (CR) centre.

## Methods

### Design and Context

This cross-sectional study conducted in the CR department of Imam Ali hospital of Kermanshah (Western part of Iran) during April 2016 to November 2016. The statistical population concluded all coronary artery bypass graft (CABG) and valve replacement patients who participated in the CR programme.

### Sample, Sampling and Sample Size

Firstly, using consecutive sampling, 194 patients participated in CR were selected. Then, 156 cases remained after screening the patients by inclusion criteria. Inclusion criteria include fluency in Farsi language, aged 20 years–80 years, ability to recall and report the emotional content of dream after cardiac surgery, and a tendency to participate. The reasons for excluding patients were: people with inappropriate age range (*n* = 5), because of their inability to remember dreams (*n* = 31), and unwillingness to participate (*n* = 2). According to formula (*n >* 50 *+* 8 m) and 10 predictor variables in this study, the sample size of more than 130 cases seems appropriate (16). Informed consent was obtained from all individual patients included in the study. Also, all procedures performed in the study were following the ethical standards of the institutional research committee and with the 1964 Helsinki declaration.

### Procedure

The steps of research were as follows: at first, the patients with criteria were identified by the research team and they were entered after written consent form and insurance for confidential identity. One week before the first session of exercise, demographic data (age, sex and education level), clinical data (anxiety, depression, anger and emotional content of dream) and medical data were recorded and collected by a clinical psychologist and a cardiologist of CR team. Beck depression and anxiety inventories, anger subscale of Buss and Perry’s aggression questionnaire and Schredl’s dream emotions manual provided to the patients by the psychologist and they fulfilled them individually after receiving necessary explanations. Also, the cardiologist recorded the data related to smoking, hypertension, diabetes mellitus, hyperlipidemia and family history of cardiac disease after an interview and assessment of patients’ medical histories.

### Instruments

#### Socio-demographics and risk factors

The samples self-reported their age, sex and current educational level in three categories: under diploma, diploma and academic degree. Also, self-reported family history of cardiac disease, smoking, hypertension, diabetes mellitus and hyperlipidemia was checked by the cardiologist.

#### The Beck anxiety inventory (BAI)

The scale is a 21-item exam of 3 scores for each item. The score of this questionnaire is varied from 0 to 63. The final score includes 4 levels of anxiety: i) score 0–7 is equal to no anxiety; ii) score 8–15 is equal to mild anxiety; iii) score 16–25 is equal to moderate anxiety; and iv) score 26–63 is equal to severe anxiety. Cronbach’s alpha of the inventory is 0.92, the credential using the retest method with a one-week interval is 0.75 and the consistency of the items is varied from 0.30 to 0.76. Validity types of this scale have also been confirmed (17). The reliability and validity of this tool have been confirmed in the Iranian population (18).

#### The Beck depression inventory (BDI)

The inventory is a 21 items exam of 3 scores for each item. The score of this questionnaire is varied from 0 to 63. Interpreting the results is determined as 5 levels: i) 0–4 means possible denial; ii) 5–9 is equal to very mild depression; iii) 10–18 is equal to mild to moderate depression; iv) 19–29 means moderate to severe depression, and v) 30–63 shows that the patients suffer from severe depression. Beck et al. (19) discovered the retest reliability in a one-week interval as 0.93. The reliability and validity of this tool have been confirmed in the Iranian population (20).

#### Buss and Perry’s aggression questionnaire (BPAQ)

It is a 29-item questionnaire where participants rank certain statements along with a 5-point continuum from ‘extremely uncharacteristic of me’ to ‘extremely characteristic of me.’ The scale has four subscales includes anger, hostility, and verbal and physical aggression. The scores are normalised on a scale of 0–1, with 1 being the highest level of aggression (21). Retest reliability of the scale in the Iranian population is 0.78. Also, the instrument validity was approved by factor analysis (22). In the current study, we used only anger subscale to assess patients. The subscale included 7 items (1-9-12-18-19-23-28) and the sum score is ranged 7–35. Buss and Perry have report Cronbach’s alpha of the anger subscale 0.83 (21).

#### Schredl’s dream emotions manual

This scale involves a dream with any positive or negative emotion (23). The emotional content intensity (both positive and negative emotions) is graded using the Likert spectrum in the 4-point category scale including none = 1, mild = 2, moderate = 3, or strong positive or negative emotions = 4. Inter-rater reliabilities for these scales in previous studies were 0.82 for negative emotions and 0.64 for positive emotions (24). In the current study, the patients divided into two groups: i) those who’s negative dream score is higher than a positive dream = 1 and ii) those who’s positive dream score is higher than a negative dream = 0.

### Data Analysis

The data related to sex, education level, family history of cardiac disease, smoking, hypertension, diabetes mellitus, hyperlipidemia and emotional content of dream were reported in a percentage format while data related to age, anxiety, depression, and anger were reported in a format of mean and standard deviation (SD). At baseline, Chi-squared and independent *t*-test were used to compare non-continuous and continuous between two groups. The binary logistic regression analysis was used to assess the relationship between all continuous and discontinuous components with the negative emotional content of the dream after an approved lack of roll-out from needed statistical pre-assumptions (16). Two models were designed and run, separately. In models 1 and 2, an adjustment was done for age × sex and for age × sex × education level, respectively. Hosmer-Lemeshow test used to evaluate the goodness of fit for logistic regression models. The Wald parameter was also reported. Analysis conducted by SPSS20 (IBM Corporation, Armonk NY, USA) for Windows and the level of significance was set at alpha *<* 0.05.

## Results

### Baseline Risk Factors and Psychosocial Components

Among the total 156 patients (64.1% male) who participated in the study, 39 patients (25%) had emotionally negative dreams. The risk factors and psychosocial characteristics of samples are presented generally and separately in Table 1. It can be seen from the table that there is a difference between the two groups in terms of anxiety, anger and hypertension. This means that anxiety and anger patients and people with hypertension history have significantly more negative dreams (*P* < 0.05), when compared to other patients,

### The Binary Logistic Regression Model

Hosmer-Lemeshow test indicated that the fit of the model is appropriate (*P* = 0.414) and our model correctly predicts 75% membership of the group. Overall, the model was statistically significant (*P* = 0.012). Indicators of effect size demonstrated suitable explanatory power concerning negative dreams. Therefore, it suggests that our model can explain 16%–23.7% variance of negative dreams. The adjusted odds ratio (adj OR), 95% confidence interval (CI) and *P*-value for each covariate are presented in Table 2. Concerning the logistic regression model, the four variables were found to be independently and significantly associated with negative dreams. According to the results, academic education level (OR = 0.25 [0.06–0.99], Wald = 3.868, *P* = 0.049), anxiety (OR = 1.08 [1.01–1.15], Wald = 4.822, *P* = 0.028), anger (OR = 1.03 [1.00–1.06], Wald = 3.961, *P* = 0.047) and hypertension (OR = 2.52 [1.05–6.02], Wald = 4.329, *P* = 0.037) have more possibility of predicting negative dreams when compared to others.

In Table 3, adjustment for demographic variables (age, sex, and education level) was conducted and the significance of every variable was assessed in two models. In Model 1, after adjustment for age and sex, it was indicated that education level, anxiety and anger can predict negative dreams (*P* < 0.05). In Model 2, after adjustment for age, sex and education, it was indicated that only anxiety (adj OR = 1.08 [1.01–1.16], *P* = 0.020) and anger (adj OR = 1.03 [1.00–1.06], *P* = 0.024) and hypertension (adj OR = 2.71 [1.10–6.68], *P* = 0.030) can predict negative dreams. Specifically, the factors can increase the negative dreams at about 1.08, 1.03 and 2.71 times, respectively.

## Discussion

The present study aimed to assess the physiological and psychological correlates of dreams with negative emotional content in patients with cardiovascular problems. The results showed that 25% of patients have negative emotional content. Hartman suggested that dreams are reflexes of life emotions during awaking time (25) and if one emotion is experienced more severe there is more possibility that it will occur in the dream (26). Patients with cardiovascular problems experience different emotions and challenges after confronting with disease and cardiac surgery (27). On the other hand, these patients usually act poorly in strategies of cognitive emotion regulation in awaking time (28). So, it seems that sleep time is the most appropriate time to regulate emotions. According to the model of the function of emotional regulation of dream, if patients experience the negative experiences in awaking time as negative emotions in a dream, they can deal with these emotions more easily (29, 30).

Another finding suggested that increased anxiety and anger and hypertension are related to the negative content. In the present study, the mean score of anger and anxiety is higher than the normal level in almost of patients. Regarding the previous reports which indicated that there is a relationship between psychological disorders and emotional content of the dream (4, 6, 7), it is suggested that negative emotional content of dream probably is a reflex of the developmental process of the psychopathology of psychiatric disorders such as anxiety and anger after diagnosis of cardiac disease (4, 6). Obviously, psychological and psychiatric symptoms manifest in patients after stabilised cardiac disease and regarding poor strategies of emotional regulation (28), the dreams with negative emotional content are responsible for emotional regulation (29). Despite this issue, lack of early treatment of anger and anxiety after a cardiac event may disrupt the quality of sleep (31, 32) so the possibility of emotional regulation will be destroyed even during sleep.

Finally, we can refer to the relationship between psychiatric disorders such as anger and anxiety with hypertension in the relationship between hypertension and negative dreams (33). The results of the previous studies indicate that inappropriate sleep quality, impulsivity, trait anxiety and dream anxiety are more in patients with hypertension compared to healthy controls (33, 34). Also, anxiety and anger induced-arousal can predict hypertension in a long-time (35, 36). So, regarding that arousal mechanism of these psychiatric components is related to hypertension in patients with cardiovascular problems, the relationship between hypertension and negative emotional content is possible.

It is noteworthy that the present results did not confirm the relationship between depression and negative emotional content. This finding is consistent with the results of a recent study by Komasi et al. (13). The findings of these researchers showed that depression alone not able to explain the negative content of dreams (13). This may be due to depression or taking antidepressants (37). Another study (38) points out that failure in recalling dreams is characterised by small EEG shifts from sleep to wakefulness in patients with depression.

The spectrum of cardiovascular diseases and interventions is very wide, including myocardial infarction (MI), coronary artery disease (CAD), chronic heart failure (VHF), pectoris angina, CABG, heart valve replacement, percutaneous coronary intervention (PCI), etc. However, in the current study, only patients undergoing CABG or heart valve replacement referred to the CR were included in the assessment. Also, data were collected only from one intervention centre using consecutive sampling resulted from the small size of the statistical population. These limitations can lead to biased results (39). So, for future studies, we recommend a selection of randomly large sample in several centres across the country.

## Conclusion

The increasing rates of anxiety and anger and history of hypertension are related to increasing dreams with negative and annoying emotional load in patients with cardiovascular problems. Although negative dreams may be effective in patients’ emotional regulation after stabilising cardiac disease, control and early treatment of psychiatric disorders and cardiac risk factors especially hypertension are concerned as necessities of secondary prevention.

## Figures and Tables

**Table 1:** Baseline risk factors and psychosocial characteristics in the overall population and in those with negative and positive dream

Variables	Overall population *N* = 156	Negative dream *n* = 39 (25%)	Positive dream *n* = 117 (75%)	*P*-value
Sex (%)^a^				0.441
Male	100 (64.1)	23 (59.0)	77 (65.8)	
Female	56 (35.9)	16 (41.0)	40 (34.2)	
Age, Mean (SD)^b^	59.1 (9.0)	61.1 (7.8)	58.5 (9.2)	0.112
Education degree (%)^a^				0.193
Under diploma	95 (60.9)	28 (71.8)	67 (57.3)	
Diploma	31 (19.9)	7 (17.9)	24 (20.5)	
Academic level	30 (19.2)	4 (10.3)	26 (22.2)	
Psychological factors, mean (SD)^b^				
Anxiety	32.72 (9.10)	35.84 (6.71)	31.46 (9.68)	**0.010**
Depression	16.24 (3.91)	16.49 (3.23)	16.14 (4.20)	0.635
Anger	63.14 (18.91)	68.43 (19.43)	60.74 (18.19)	**0.027**
Risk factors (%)^a^				
Diabetes	32 (20.5)	6 (15.4)	33 (22.2)	0.360
Hypertension	63 (40.4)	22 (56.4)	41 (35.0)	**0.019**
Hyperlipidemia	45 (28.8)	12 (30.8)	33 (28.2)	0.760
Family history	90 (57.7)	19 (48.7)	71 (60.7)	0.190
Smoking	63 (40.4)	13 (33.3)	50 (42.7)	0.462

Notes: Significant difference between patients with positive and negative dream for each character is as:

* *P* < 0.05

a Chi-squared test;

b *t*-test

**Table 2:** Correlates of negative dream in the overall population (univariate model)

Variables	Negative dream (%)	Odds ratio (OR)	Wald	*P*-value
Sex (%)				
Male	23.0	Referent		
Female	28.6	1.21 (0.43–3.44)	0.131	0.718
Age (M ± SD)	-	1.03 (0.98–1.09)	1.317	0.251
Education level (%)				
Under diploma	29.5	Referent		
Diploma	22.6	0.86 (0.30–2.51)	0.077	0.782
Academic level	13.3	0.25 (0.06–0.99)	3.868	**0.049**
Psychological factors (M ± SD)				
Anxiety	-	1.08 (1.01–1.15)	4.822	**0.028**
Depression	-	0.89 (0.76–1.05)	1.903	0.168
Anger	-	1.03 (1.00–1.06)	3.961	**0.047**
Risk factors (%)				
Diabetes	18.7	0.74 (0.25–2.15)	0.313	0.576
Hypertension	34.9	2.52 (1.05–6.02)	4.329	**0.037**
Hyperlipidemia	26.7	1.04 (0.40–2.69)	0.007	0.933
Family history	21.1	0.62 (0.27–1.43)	1.279	0.258
Smoking	20.6	0.72 (0.29–1.83)	0.469	0.494

Notes: The risk factors and psychosocial characteristics listed in this table were all included as covariates in the generation of the binary logistic regression model

Statistically significant odds ratio for each character is * *P* < 0.05

**Table 3:** Correlates of negative dream after adjusted for demographics

Variables	Model 1				Model 2			

	adj OR	95% CI	Wald	*P*-value	adj OR	95% CI	Wald	*P*-value
Education	0.24	0.06–0.96	4.043	**0.044**	4.48	0.31–64.12	1.223	0.269
Anxiety	1.08	1.01–1.15	5.120	**0.024**	1.08	1.01–1.16	5.420	**0.020**
Anger	1.03	1.00–1.06	3.762	**0.047**	1.03	1.00–1.06	5.116	**0.024**
Hypertension	2.42	1.01–5.81	3.930	0.052	2.71	1.10–6.68	4.681	**0.030**

Notes: Model 1: adjusted for age and sex. Model 2: adjusted for age, sex and education level
